# 1747. Clinical Spectrum of a Large Multi-State U.S. Cohort of Parechovirus Infection in Neonates and Young Infants

**DOI:** 10.1093/ofid/ofad500.1578

**Published:** 2023-11-27

**Authors:** Amanda S Evans, Laura Filkins, Haidee Custodio, Elizabeth A Daniels, Carol Kao, Katherine M Richardson, Maria Carrillo-Marquez, Carla Isabel Borré, Carlos R Oliveira, Claudia M Espinosa, Joseph A Bocchini, Yamini Mandelia, Marc A Mazade, Roberto P Santos, Silvana Carr, Rangaraj Selvarangan, David W Kimberlin

**Affiliations:** UT Southwestern Medical Center, Dallas, Texas; University of Texas Southwestern/Children's Health, Dallas, Texas; University of South Alabama College of Medicine, Mobile, Alabama; Washington University in St. Louis School of Medicine, St. Louis, Missouri; WASHINGTON UNIVERSITY IN ST LOUIS SCHOOL OF MEDICINE, ST LOUIS, Missouri; Prisma Health, Greenville, South Carolina; University of Tennessee Health Science Center College of Medicine, Memphis, Tennessee; Yale School of Public Health, Porto Alegre, Rio Grande do Sul, Brazil; Yale University, New Haven, Connecticut; MORSANI COLLEGE OF MEDICINE PEDIATRICS, Tampa, Florida; Tulane University, Shreveport, Louisiana; ECU Health- Brody School of Medicine, Greenville, North Carolina; Cooks Childrens Physician Network, Fort Worth, TX; University of Mississippi Medical Center, Jackson, Mississippi; Children's Mercy Hospital, Kansas, Missouri; Children’s Mercy Kansas City, Kansas City, Missouri; University of Alabama at Birmingham, Birmingham, Alabama

## Abstract

**Background:**

Severe manifestations of human parechovirus (HPeV) infection, including meningoencephalitis and sepsis, can occur in neonates and young infants. Death has rarely been reported. Rates of HPeV infection fluctuate from year to year in the community. After the home isolation during the initial COVID-19 era in 2020, waves of respiratory viral outbreaks at unusual times of the year were identified in the pediatric community in 2021 and 2022, including increased number of cases of severe HPeV infection in young infants. This study characterizes the U.S. outbreak of HPeV infections that occurred in newborns and young infants in 2022.

**Methods:**

A multi-site, retrospective and prospective observational study identified hospitalized infants < =6 months, with a body fluid specimen positive for HPeV. IRB approval of a single protocol was obtained at each site. Data obtained included hospital summary, maternal and birth history, medications, bacterial and viral testing, and radiology results. Analysis included infants with hospitalizations from January 1 through December 31, 2022.

**Results:**

13 academic sites were enrolled, which included 8 sites achieving full IRB approval/activation and completed data collection, and 5 sites pending activation [Fig 1A]. Interim analysis shows 112 infants across eleven states hospitalized with HPeV infection with a median hospital duration of 3 days (range 0-178 d), peaking in May 2022 [Fig 1B], with a spectrum of biochemical markers [Table 1]. The median age at hospitalization was 21.5 days of life (IQR 10, 32 d). All but five infants received antibiotics during the admission, and more than half (54%) received acyclovir. Other pathogens were rarely reported [Table 2]. 45 (38%) infants received MRI brain imaging during hospitalization of which 29 were abnormal; 10 reports directly mentioning HPeV in the interpretation. A total of 51 EEGs were completed in 31 infants; 20 infants (18%) had seizures reported. 17 infants required anticonvulsants. Two infants died.

**Figure:**
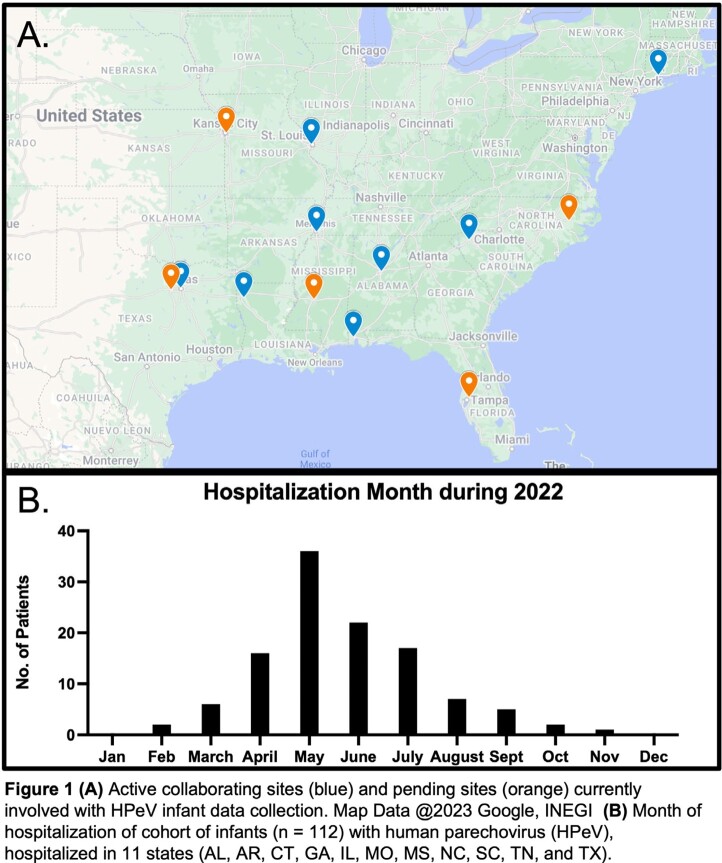

**Figure:**
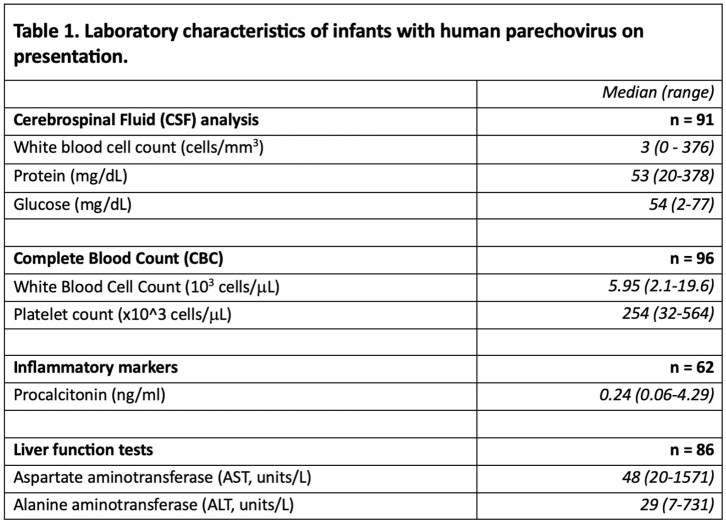

**Figure:**
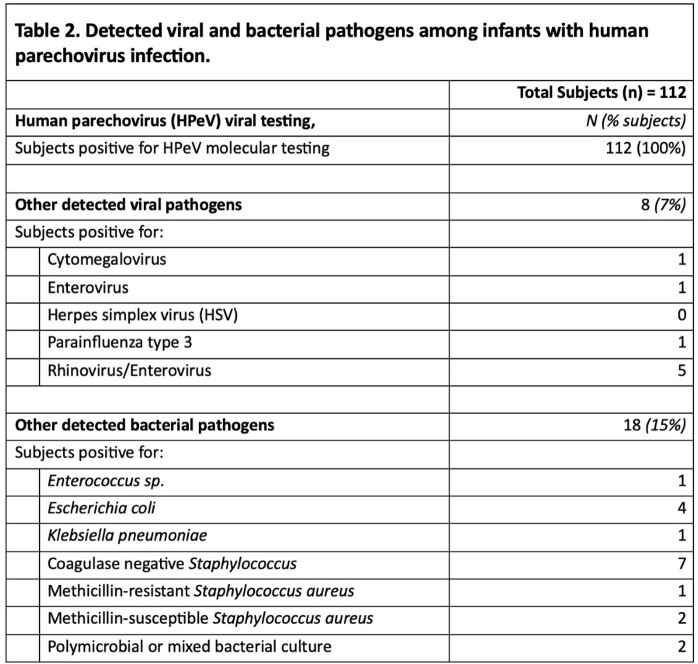

**Conclusion:**

We report the first multi-site, clinical description of the 2022 U.S. outbreak of HPeV among neonates and young infants. This is the first large series of infants with HPeV to report mortality. Further development of a multi-site U.S. collaboration to improve surveillance for HPeV is needed.

**Disclosures:**

**Laura Filkins, PhD**, Avsana Labs: Board Member|Avsana Labs: Stocks/Bonds|Biofire Diagnostics: Grant/Research Support **Claudia M. Espinosa, MD, MSc**, AstraZeneca: Grant/Research Support|Enanta: Grant/Research Support|Jansen and Jansen: Advisor/Consultant|Kentucky Rural Health Association: Honoraria|Merck: Grant/Research Support|Sanofi: Advisor/Consultant|Sanofi: Grant/Research Support|Sanofi: Honoraria|Sobi: Dinner **Joseph A. Bocchini, Jr., MD**, Avalere: Advisor/Consultant|Enanta: Site PI on multicenter clinical trials, contract with employer|Moderna: Advisor/Consultant|Novavax: Sub-I on multicenter clinical trials, contract with employer|Pfizer: Advisor/Consultant|Pfizer: Site PI on multicenter clinical trials, contract with employer|Regeneron: Site PI on multicenter clinical trials, contract with employer|Sobi: Advisor/Consultant|Valneva: Advisor/Consultant **Roberto P. Santos, MD, MSCS**, Eli Lilly (SARS-CoV-2, baricitinid) - Site Open for Enrollment on March 8, 2023: Industry-sponsored clinical trials with research contract awarded to the University of Mississippi Medical Center with RPS as site PI|Eli Lilly (SARS-CoV-2, LY3819253, LY3832479) - study discontinuation on January 3, 2022: Industry-sponsored clinical trials with research contract awarded to the University of Mississippi Medical Center with RPS as site PI|JNJ (RSV, rilematovir) - study discontinuation on March 1, 2022: Industry-sponsored clinical trials with research contract awarded to the University of Mississippi Medical Center with RPS as site PI|MSD (HIV, islatravir, doravirine) - study discontinuation on December 6, 2022: Industry-sponsored clinical trials with research contract awarded to the University of Mississippi Medical Center with RPS as site PI **Rangaraj Selvarangan, BVSc, PhD, D(ABMM), FIDSA, FAAM**, Abbott: Honoraria|Altona Diagnostics: Grant/Research Support|Baebies Inc: Advisor/Consultant|BioMerieux: Advisor/Consultant|BioMerieux: Grant/Research Support|Bio-Rad: Grant/Research Support|Cepheid: Grant/Research Support|GSK: Advisor/Consultant|Hologic: Grant/Research Support|Lab Simply: Advisor/Consultant|Luminex: Grant/Research Support **David W. Kimberlin, MD**, Gilead: Grant/Research Support|Gilead: I served as site PI on Gilead's study of remdesivir in pediatric patients. All monies went directly to my university and not to me.

